# Predictive value of heart rate for prognosis in patients with cerebral infarction without atrial fibrillation comorbidity analyzed according to the MIMIC-IV database

**DOI:** 10.3389/fneur.2025.1551427

**Published:** 2025-03-14

**Authors:** Xinrou Song, Luwen Zhu

**Affiliations:** ^1^Department of Rehabilitation Medicine, Heilongjiang University of Chinese Medicine, Harbin, China; ^2^The Second Affiliated Hospital of Heilongjiang University of Chinese Medicine, Harbin, China

**Keywords:** MIMIC-IV database, cerebral infarction, heart rate, prognosis, predictive modeling

## Abstract

**Objective:**

This study focused on the relationship between heart rate and the likelihood of death within 28 days in patients with cerebral infarction without the comorbidity of atrial fibrillation, using patient data extracted from the MIMIC-IV database.

**Method:**

This study involved a retrospective analysis of clinical data from 1,643 individuals with cerebral infarction who were admitted to the ICU. To investigate the role of heart rate in determining patient survival, we applied a variety of statistical techniques such as Cox regression models, survival analysis using Kaplan–Meier plots, and spline-based models. In addition, we performed analyses by patient subgroups to identify any potential variables that could influence the association between HR and 28-day mortality.

**Result:**

In univariate and multivariate analyses, elevated heart rate was strongly associated with higher 28-day mortality, even after adjusting for confounders such as age, sex, comorbidities, and clinical scores.(HR:1.01, 95%,CI:1.01 ~ 1.02, *p* = 0.019) Kaplan–Meier survival analysis showed that patients with heart rate > 90 beats/min had a significantly lower probability of survival. Restricted cubic spline (RCS) analysis confirmed a nonlinear relationship between heart rate and mortality. Subgroup analyses demonstrated an interaction between heart rate and factors such as hypertension and mechanical ventilation status.

**Conclusion:**

This study highlights the prognostic significance of heart rate as an independent predictor of 28-day mortality in patients with cerebral infarction who do not have atrial fibrillation.

## 1 Introduction

Cerebral infarction continues to be a major contributor to global death rates and prolonged disability, imposing considerable strain on healthcare systems and society as a whole. As the population ages and the prevalence of cardiovascular risk factors like hypertension, diabetes, and hyperlipidemia increases, its incidence is anticipated to grow worldwide (1). Despite advances in acute-phase management and secondary prevention strategies, the prognosis for many patients with cerebral infarction remains poor, is often comorbid with cardiovascular disease, and is highly variable depending on individual clinical and physiologic factors (2, 36). For instance, studies focusing on young and middle-aged cerebral infarction patients have identified several independent risk factors for poor prognosis. Among the variables being examined are the severity of neurological impairment assessed through stroke scales, variations in the MTHFR gene, and the condition of elevated blood pressure (3). A one-year follow-up study of 323 patients with acute cerebral infarction revealed that age, NIHSS score at admission, diabetes, and hypertension are key determinants of poor prognosis (4).

Heart rate is a fundamental physiological parameter that reflects the rhythmic contractions of the heart per minute. It is regulated by the autonomic nervous system through the interaction of sympathetic and parasympathetic inputs, maintaining cardiovascular balance (5). In addition to being a marker of cardiovascular function, heart rate is also an important indicator of systemic pressure, metabolic demand, and overall autonomic nervous system balance. Changes in heart rate can provide valuable information about an individual’s hemodynamic and neurological status, especially during acute illness (6). Heart rate variability is significantly reduced in patients with acute cerebral infarction, indicating impaired autonomic nervous system regulation (7). Reduced heart rate variability is linked to an increased risk of mortality and poorer outcomes in individuals experiencing myocardial infarction (8). These findings underscore the potential value of heart rate as an indicator of disease severity and recovery progression across different conditions. Nonetheless, the connection between heart rate and prognosis in patients with cerebral infarction is still not well defined. Some physiological mechanisms, such as systemic inflammation (9) and autonomic dysfunction (10), may link heart rate to the prognosis of cerebral infarction. Although some studies suggest that both tachycardia and bradycardia may influence mortality and neurological recovery (11–13). A retrospective cohort study explored the association between mean heart rate and 30-day mortality in ischemic stroke patients with atrial fibrillation, who were admitted to the intensive care unit within 24 h of hospital arrival. The findings revealed a J-shaped correlation between mean heart rate and 30-day mortality (14). However, there is a lack of strong evidence regarding the connection between heart rate and prognosis in patients with cerebral infarction who do not have atrial fibrillation, leaving the prognostic value of heart rate in this group unclear.

The purpose of this investigation was to assess the potential impact of heart rate on short-term survival outcomes (particularly 28-day mortality) in patients with cerebral infarction who do not have atrial fibrillation. To perform this study, we rely on data extracted from the Medical Information Mart for Intensive Care-IV (the MIMIC-IV) database, which compiles a wide range of clinical information from ICU patients, allowing for a comprehensive evaluation of this relationship. By elucidating the prognostic value of heart rate in cerebral infarction patients, our findings will provide valuable insights for risk stratification and management strategies in clinical practice.

## 2 Materials and methods

### 2.1 Data source

The MIMIC-IV database is a publicly available, large-scale intensive care unit (ICU) database maintained by the MIT Lab for Computational Physiology (15). This collection features anonymized health records for individuals who received treatment in the ICU at a major medical facility in Boston, Massachusetts, spanning from 2008 through 2019. The dataset includes detailed information on demographics, vital signs, laboratory tests, medications, surgeries, and clinical outcomes, enabling strong epidemiological and clinical investigations. Author Xinrou Song has successfully completed the required training and certification for accessing the MIMIC-IV database (Certificate No. 66625234) and was responsible for extracting the data used in this study.

### 2.2 Data extraction, patient grouping, and outcome measures

The data for this study were extracted from the MIMIC-IV database, which contains de-identified ICU patient information. Data extraction was performed using PostgreSQL software, and to ensure data consistency and relevance to the acute phase, only data from the first 24 h after patient admission were included.

These data points encompassed: biological sex, age group, racial background, blood parameters such as hematocrit, hemoglobin, platelet count, red cell distribution width (RDW), electrolyte balance (e.g., calcium, chloride, sodium), metabolic markers like blood urea nitrogen (BUN), creatinine, international normalized ratio (INR), prothrombin time (PT), and partial thromboplastin time (PTT) and glucose, as well as acid–base balance indicators (anion gap and bicarbonate). Furthermore, clinical factors covered heart rate, blood pressure (systolic, diastolic, and mean arterial), temperature, and specific medical conditions including heart failure, coronary artery disease, peripheral artery disease, neurological conditions like dementia and paraplegia, pulmonary disorders, liver dysfunction, renal impairment, metabolic diseases such as diabetes and hyperlipidemia. The study also incorporated clinical assessment tools, including SOFA, GCS, which helped in evaluating the severity of patient conditions. Laboratory values were recorded as minimum values, while vital signs (e.g., heart rate) were recorded as averages. The outcome was defined as whether the patient survived or died within 28 days of admission. Patients were divided into two groups: the 28-day survival group and the 28-day mortality group.

### 2.3 Exclusion criteria

This study investigated the impact of heart rate on the prognosis of stroke patients, using the MIMIC-IV database. Eligibility criteria required patients to have been admitted to the ICU for the first time and diagnosed with cerebral infarction, based on the ICD-9 and ICD-10 criteria.

**Figure 1:**
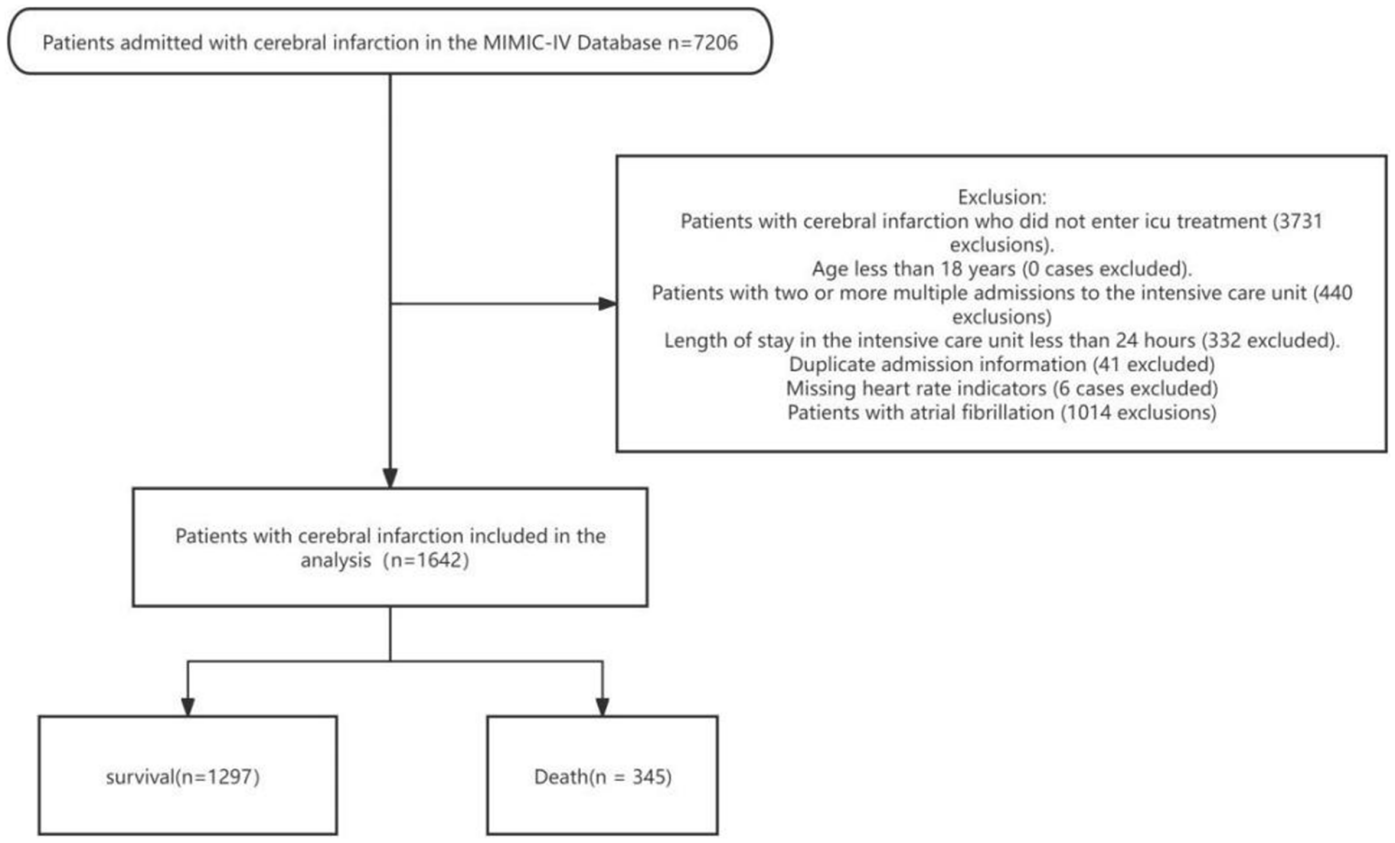
Flow-diagram illustrating patient inclusion in the study.

The following exclusion criteria were applied: The inclusion criteria for this study were patients diagnosed with cerebral infarction who received ICU treatment in the MIMIC-IV database. Exclusion criteria included patients who did not undergo ICU treatment, those with multiple ICU admissions, an ICU stay of less than 24 h, duplicate admission records, missing heart rate data, and a history of atrial fibrillation. Additionally, patients under the age of 18 were excluded from the analysis (Figure 1). To reduce potential bias, variables with missing data exceeding 15% were excluded from the analysis. For variables with less than 15% missing data, missing values were imputed using the random forest method from the missForest R package. This approach helped maintain group homogeneity and enhanced the reliability of the subsequent analysis.

### 2.4 Statistical analysis

All analyses were performed using R software (version 4.2), along with appropriate data handling and modeling tools. The normality of continuous variables was initially assessed using the Shapiro–Wilk test. Variables that followed a normal distribution were expressed as mean ± standard deviation, and group differences were assessed using the independent t-test. For non-normally distributed data, results were presented as the median with interquartile range (P_25_, P_75_). Categorical data were presented as frequencies and percentages. The Wilcoxon rank-sum test was used to compare continuous variables across groups, while categorical variables were analyzed using the Pearson chi-square test.

To explore the relationship between heart rate and risk of death, we used a Cox proportional hazards regression model to calculate hazard ratios (HR) and 95% confidence intervals (CI). A multivariate model was then constructed to account for potential confounders. Variables with *p*-values less than 0.05 on univariate analysis were included in the final multivariate model. To assess the relationship between heart rate and in-hospital mortality, four separate models were constructed.

To identify the most relevant heart rate threshold associated with in-hospital mortality, ROC curve analysis was performed, with the Youden index used to determine the optimal cutoff value. This threshold was then applied to divide patients into two groups: one with a low heart rate and the other with a high heart rate. The difference in 28-day survival probabilities between the two groups was assessed using Kaplan–Meier survival curves, followed by a log-rank test. Additionally, non-linear relationships between heart rate and mortality risk were explored using restrictive cubic spline (RCS) analysis. Subgroup analyses were carried out to evaluate the potential moderating effects of gender, age, and comorbidities on the association between heart rate and mortality risk. Interaction terms were incorporated into the models to assess statistical significance, with interaction *p*-values reported. All statistical tests were two-sided, and a *p*-value of less than 0.05 was considered statistically significant. Effect sizes and confidence intervals were provided to emphasize the clinical relevance of the findings.

## 3 Results

### 3.1 Baseline characteristics of two groups of stroke patients

All participants were categorized into survival and death groups. Their baseline characteristics are summarized in Table 1. The non-survivor group was older (*p* < 0.001) and had a higher proportion of “other” in the racial distribution (*p* < 0.001). Their white WBC, anion gap, BUN, creatinine, glucose, RDW, INR, and PT were elevated (*p* < 0.05) in the mortality group, whereas hematocrit, hemoglobin, platelets, and bicarbonate were elevated (*p* < 0.05) in the surviving cohort. Vital signs showed higher heart rate and respiratory rate, lower DBP, MBP, and temperature (*p* < 0.05), higher SOFA score, and lower GCS score (*p* < 0.05) in the deceased cohort. SBP was higher in the survivor group (*p* = 0.023) Comorbidities such as myocardial infarction, heart failure, dementia, lung disease, liver disease, renal disease and hypertension were more common in the mortality group (*p* < 0.05) (Table 1).

**Table 1:** Baseline characteristics of all participants according to survival status.

Characteristics	Total (*n* = 1,642)	Survival (*n* = 1,297)	Death (*n* = 345)	*P*
Age	66.00 (54.00, 76.00)	64.00 (53.00, 74.00)	72.00 (61.00, 82.00)	<0.001
Gender (years)				0.206
Female	788 (47.99)	612 (47.19)	176 (51.01)	
Man	854 (52.01)	685 (52.81)	169 (48.99)	
Race				<0.001
White	1,024 (62.36)	833 (64.23)	191 (55.36)	
Black	209 (12.73)	172 (13.26)	37 (10.72)	
Others	409 (24.91)	292 (22.51)	117 (33.91)	
Laboratory tests				
Hematocrit	34.50 (29.10, 38.80)	35.00 (29.80, 39.30)	32.50 (27.10, 37.10)	<0.001
Hemoglobin (g/L)	11.50 (9.50, 13.00)	11.70 (9.80, 13.10)	10.70 (8.80, 12.10)	<0.001
Platelets (×10^9^/L)	198.50 (151.25, 253.00)	201.00 (156.00, 256.00)	183.00 (128.00, 246.00)	<0.001
WBC (×10^9^/L)	9.30 (7.20, 12.30)	9.00 (7.10, 11.70)	10.70 (7.80, 15.10)	<0.001
Aniongap (mEq/L)	13.00 (12.00, 15.00)	13.00 (12.00, 15.00)	13.00 (12.00, 16.00)	0.032
Bicarbonate (mEq/L)	22.00 (20.00, 24.00)	22.00 (20.00, 24.00)	21.00 (18.00, 24.00)	<0.001
BUN (mg/dL)	15.00 (11.00, 22.00)	15.00 (10.00, 21.00)	17.00 (13.00, 28.00)	<0.001
Calcium (mEq/L)	8.50 (7.90, 8.90)	8.60 (8.00, 9.00)	8.20 (7.70, 8.60)	<0.001
Chloride (mEq/L)	103.00 (100.00, 106.00)	103.00 (100.00, 106.00)	103.00 (100.00, 107.00)	0.914
Creatinine (mg/dL)	0.90 (0.70, 1.17)	0.80 (0.70, 1.10)	0.90 (0.70, 1.40)	0.002
Glucose (mg/dl)	114.00 (96.00, 140.00)	111.00 (95.00, 137.00)	124.00 (103.00, 149.00)	<0.001
RDW (%)	13.80 (13.10, 14.90)	13.70 (13.00, 14.70)	14.20 (13.40, 15.90)	<0.001
Sodium (mEq/L)	138.00 (136.00, 141.00)	138.00 (136.00, 141.00)	138.00 (136.00, 141.00)	0.278
Potassium (mEq/L)	3.85 (3.50, 4.20)	3.90 (3.50, 4.20)	3.80 (3.40, 4.20)	0.282
INR	1.10 (1.00, 1.20)	1.10 (1.00, 1.20)	1.10 (1.00, 1.30)	<0.001
PT (s)	12.20 (11.30, 13.30)	12.10 (11.30, 13.10)	12.60 (11.60, 14.40)	<0.001
PTT (s)	27.30 (25.00, 30.50)	27.30 (25.00, 30.40)	27.60 (25.00, 31.50)	0.178
Vital signs				
Heart rate (beats/min)	78.78 (69.92, 89.62)	78.16 (69.23, 88.46)	82.30 (72.41, 94.87)	<0.001
SBP (mmHg)	130.81 (117.36, 145.35)	131.52 (117.81, 145.95)	128.65 (115.72, 143.29)	0.023
DBP (mmHg)	68.45 (59.97, 78.02)	69.79 (60.50, 79.35)	64.07 (58.42, 72.33)	<0.001
MBP (mmHg)	86.16 (76.80, 94.99)	87.24 (77.50, 96.02)	82.36 (75.44, 90.94)	<0.001
Respiratory rate (beats/min)	18.61 (16.80, 21.08)	18.44 (16.70, 20.74)	19.38 (17.18, 22.61)	<0.001
Temperature (°C)	36.93 (36.72, 37.20)	36.92 (36.73, 37.16)	36.97 (36.70, 37.34)	0.024
SpO_2_ (%)	97.24 (96.00, 98.60)	97.11 (95.92, 98.42)	97.81 (96.48, 99.07)	<0.001
Score				
SOFA	3.00 (2.00, 5.00)	3.00 (1.00, 5.00)	5.00 (3.00, 8.00)	<0.001
GCS	14.00 (12.00, 15.00)	14.00 (12.00, 15.00)	14.00 (8.00, 15.00)	0.008
Comorbidities, *n* (%)				
Myocardial infarct	251 (15.29)	183 (14.11)	68 (19.71)	0.010
Congestive heart failure	283 (17.24)	207 (15.96)	76 (22.03)	0.008
Peripheral vascular Disease	206 (12.55)	168 (12.95)	38 (11.01)	0.334
Dementia	55 (3.35)	30 (2.31)	25 (7.25)	<0.001
Chronic pulmonary disease	296 (18.03)	215 (16.58)	81 (23.48)	0.003
Rheumatic disease	57 (3.47)	40 (3.08)	17 (4.93)	0.096
Peptic ulcer disease	22 (1.34)	18 (1.39)	4 (1.16)	0.949
Liver disease	118 (7.19)	80 (6.17)	38 (11.01)	0.002
Diabetes	536 (32.64)	411 (31.69)	125 (36.23)	0.110
Paraplegia	720 (43.85)	571 (44.02)	149 (43.19)	0.781
Renal disease	269 (16.38)	200 (15.42)	69 (20.00)	0.041
Hypertension	537 (32.70)	407 (31.38)	130 (37.68)	0.027
Hyperlipidemia	687 (41.84)	547 (42.17)	140 (40.58)	0.594

Values are expressed as the median (Interquartile range) or *n* (%). WBC, white blood cell; BUN, blood urea nitrogen; RDW, Red blood cell volume distribution width; INR, international normalized ratio; PT, prothrombin time; PTT, partial thromboplastin time; SBP, systolic blood pressures; DBP, diastolic blood pressures; MBP, mean arterial pressures; SpO_2_, oxygen saturation; SOFA, Sequential Organ Failure Assessment score; GCS, Glasgow coma scale.

### 3.2 Univariate cox regression analysis of risk factors for mortality in stroke patients

Table 2 summarizes factors significantly associated with mortality (*p* < 0.05). Higher mortality risk was linked to older age, ‘Others’ race, and comorbidities such as myocardial infarction, congestive heart failure, dementia, chronic pulmonary disease, liver disease, and hypertension. Laboratory abnormalities included elevated WBC, anion gap, BUN, glucose, RDW, INR, and PT, while lower hematocrit, hemoglobin, bicarbonate, platelets, and calcium were associated with increased risk. Vital sign differences included higher heart and resp. rate, SpO_2_, and lower SBP, DBP, and MBP. Additionally, higher SOFA scores and lower GCS scores were significantly related to mortality.

**Table 2:** Univariate analysis of hospital mortality.

Characteristics	HR (95%CI)	*P*
Age	1.03 (1.02 ~ 1.04)	<0.001
Race		
White	1.00 (Reference)	
Black	0.94 (0.66 ~ 1.33)	0.714
Others	1.65 (1.31 ~ 2.07)	<0.001
Myocardial infarct	1.40 (1.07 ~ 1.82)	0.014
Congestive heart failure	1.40 (1.07 ~ 1.82)	0.014
Dementia	2.61 (1.74 ~ 3.92)	<0.001
Chronic pulmonary disease	1.44 (1.12 ~ 1.84)	0.004
Liver disease	1.68 (1.20 ~ 2.35)	0.003
Renal disease	1.28 (0.98 ~ 1.66)	0.071
Hypertension	1.29 (1.03 ~ 1.60)	0.023
Hematocrit	0.96 (0.95 ~ 0.98)	<0.001
Hemoglobin	0.88 (0.85 ~ 0.92)	<0.001
WBC	1.03 (1.02 ~ 1.04)	<0.001
Aniongap	1.04 (1.01 ~ 1.08)	0.014
Bicarbonate	0.92 (0.90 ~ 0.95)	<0.001
BUN	1.01 (1.01 ~ 1.02)	<0.001
Platelets	0.99 (0.99 ~ 0.99)	0.009
Calcium	0.69 (0.61 ~ 0.77)	<0.001
Creatinine	1.03 (0.97 ~ 1.10)	0.267
Glucose	1.01 (1.01 ~ 1.01)	<0.001
RDW	1.13 (1.08 ~ 1.19)	<0.001
INR	1.62 (1.26 ~ 2.09)	<0.001
PT	1.04 (1.02 ~ 1.07)	<0.001
Heart Rate	1.02 (1.01 ~ 1.02)	<0.001
SBP	0.99 (0.99 ~ 0.99)	0.031
DBP	0.97 (0.97 ~ 0.98)	<0.001
MBP	0.98 (0.97 ~ 0.99)	<0.001
Respiratory rate	1.07 (1.04 ~ 1.09)	<0.001
Temperature	1.09 (0.87 ~ 1.35)	0.457
SpO_2_	1.15 (1.08 ~ 1.22)	<0.001
SOFA	1.15 (1.12 ~ 1.18)	<0.001
GCS	0.90 (0.87 ~ 0.92)	<0.001

HR, Hazard Ratio; CI, Confidence Interval; WBC, white blood cell; BUN, blood urea nitrogen; RDW, Red blood cell volume distribution width; INR, international normalized ratio; PT, prothrombin time; SBP, systolic blood pressures; DBP, diastolic blood pressures; MBP, mean arterial pressures; SpO_2_, oxygen saturation; SOFA, Sequential Organ Failure Assessment score; GCS, Glasgow coma scale.

### 3.3 Multivariable cox regression analysis of heart rate and mortality in stroke patients

Table 3 presents the Cox regression analysis of the relationship between heart rate variability and in-hospital mortality across four models. In all models, heart rate was significantly associated with in-hospital mortality. In the crude model (Model 1), heart rate showed a significant association (*p* < 0.001), which remained significant after adjusting for age and race in Model 2 (*p* < 0.001). Further adjustments for laboratory parameters, vital signs, SOFA, and GCS in Model 3 still demonstrated a significant association (*p* = 0.019). This association persisted in Model 4 after additional adjustments for comorbidities, including myocardial infarction, congestive heart failure, dementia, chronic pulmonary disease, liver disease, and hypertension (*p* = 0.019).

**Table 3:** Cox hazardous regression of the relationship between heart rate and 28-day mortality.

Categories	Model1		Model2		Model3		Model4	
	HR (95%CI)	*P*	HR (95%CI)	*P*	HR (95%CI)	*P*	HR (95%CI)	*P*
Heart Rate	1.02 (1.01 ~ 1.02)	<0.001	1.02 (1.01 ~ 1.03)	<0.001	1.01 (1.01 ~ 1.02)	0.019	1.01 (1.01 ~ 1.02)	0.019

HR, Hazard Ratio; CI, Confidence Interval; WBC, white blood cell; BUN, blood urea nitrogen; RDW, Red blood cell volume distribution width; INR, international normalized ratio; PT, prothrombin time; SBP, systolic blood pressures; DBP, diastolic blood pressures; MBP, mean arterial pressures; SpO2, oxygen saturation; SOFA, Sequential Organ Failure Assessment score; GCS, Glasgow coma scale. Model1: Crude. Model2: Adjust: age, race. Model3: Adjust: age, race, hematocrit, hemoglobin, platelets, WBC, aniongap, bicarbonate, BUN, calcium, glucose, RDW, INR, PT, SBP, DBP, MBP, Resp rate, SpO2, SOFA, GCS. Model4: Adjust: age, race, hematocrit, hemoglobin, platelets, WBC, aniongap, bicarbonate, BUN, calcium, glucose, RDW, INR, PT, SBP, DBP, MBP, Resp rate, SpO2, Myocardial infarct, Congestive heart failure, Dementia, Chronic pulmonary disease, Liver disease, Hypertension, SOFA, GCS*..

### 3.4 The relationship between heart rate stratification and 28-day survival rate based on Kaplan–Meier survival curves and ROC analysis

In the Kaplan–Meier survival curve analysis, heart rate stratification (high heart rate vs. low heart rate) was used to assess the 28-day survival probability. To evaluate the predictive value of heart rate for in-hospital mortality in critically ill patients with acute cerebral infarction, we plotted the receiver operating characteristic (ROC) curve. A heart rate threshold of 89.72 beats per minute (sensitivity = 35.36%, specificity = 77.95%) was used to divide patients into two groups: low heart rate (≤89.72 bpm, *n* = 1,234) and high heart rate (>89.72 bpm, *n* = 408).

**Figure 2:**
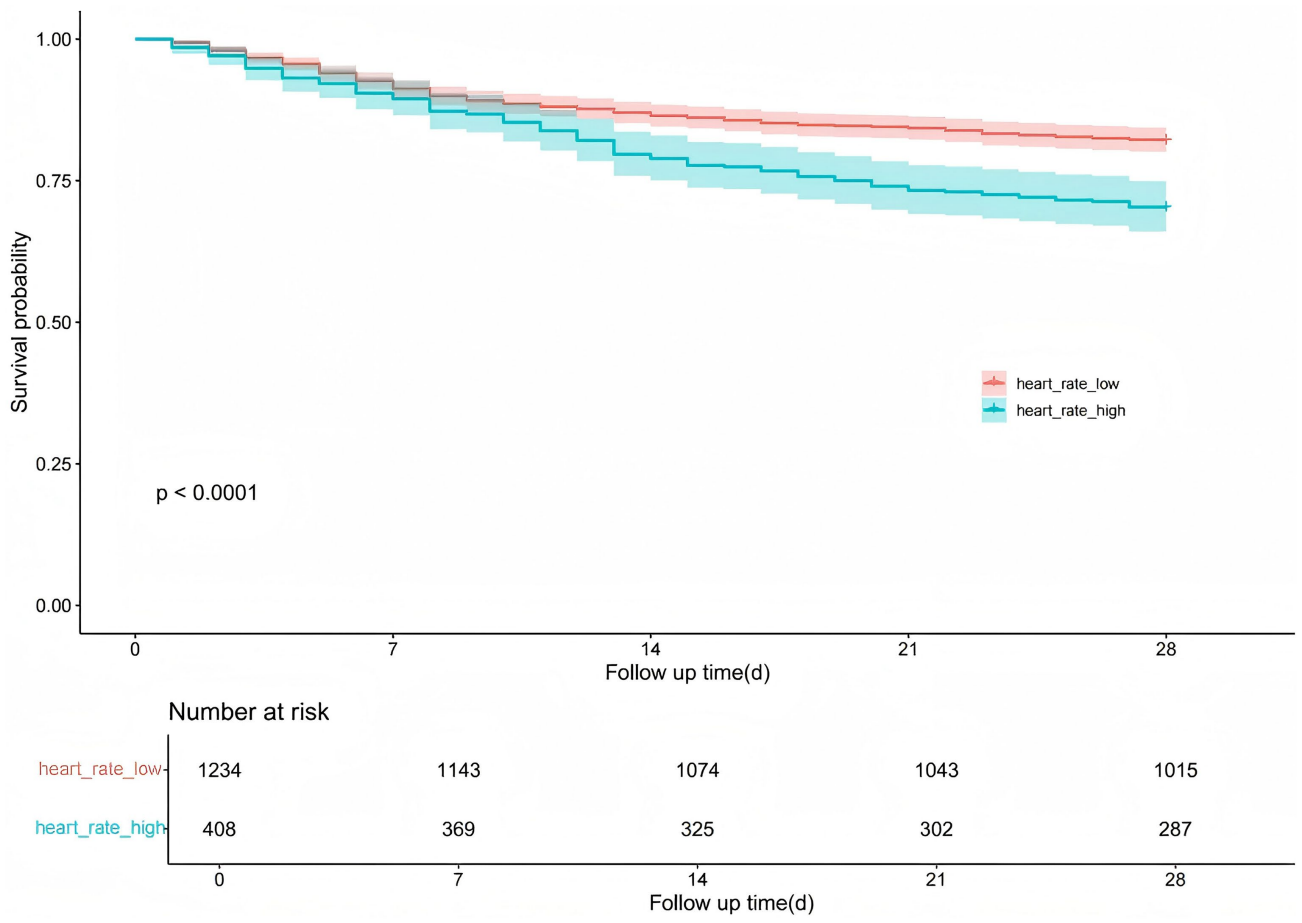
Kaplan–Meier survival curves: effect of heart rate grouping on the probability of 28-day survival in patients with cerebral infarction.

The Kaplan–Meier survival curve illustrates the association between heart rate levels and in-hospital mortality over a 28-day follow-up period. Patients were divided into two groups: high heart rate and low heart rate. The survival probability was significantly lower in the high heart rate group compared to the low heart rate group (*p* < 0.0001). The number at risk decreased consistently over time in both groups, with 1,234 and 408 patients at baseline in the low and high heart rate groups, respectively. This survival difference remained evident throughout the follow-up period, with the high heart rate group exhibiting a steeper decline in survival probability (Figure 2).

### 3.5 Non-linear relationship between heart rate and mortality risk based on RCS curve analysis

**Figure 3:**
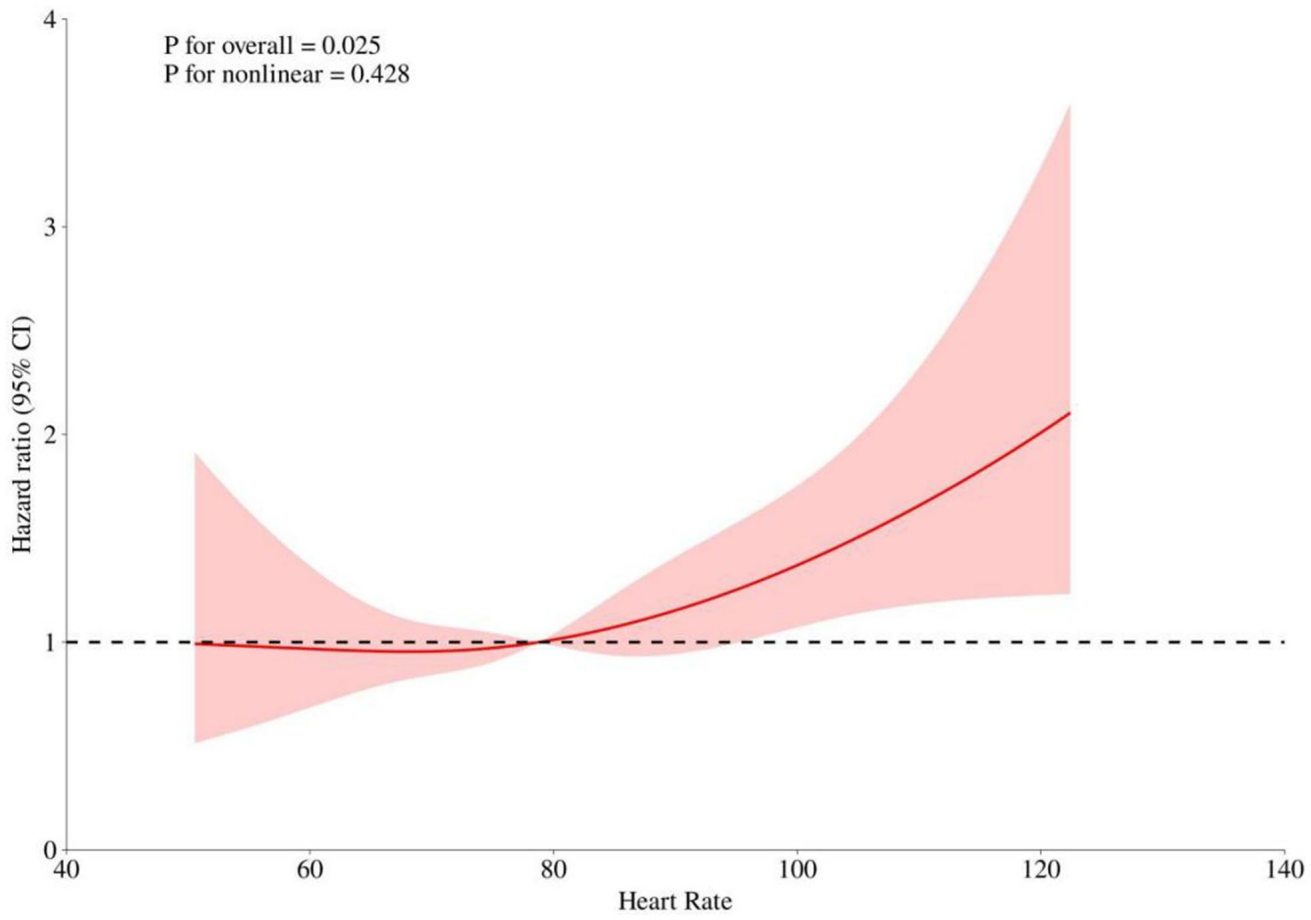
Restricted Cubic Spline (RCS) Curves Analyzing the Relationship Between Heart Rate and Risk of Death. This figure shows the overall and nonlinear relationship between heart rate and the risk ratio (HR) of death. The solid red line indicates the risk ratio fitted to the RCS model, and the shaded area indicates the 95% confidence interval. The dashed line indicates the reference line for HR = 1.

After adjusting for key covariates including age, race, hematocrit, hemoglobin, platelets, WBC, BUN, glucose, SOFA score, and GCS, RCS models were used to explore the linear relationship between heart rate and 30-day mortality risk. The analysis showed no significant non-linear association (*p* for non-linearity = 0.428). A positive correlation with increased mortality risk was observed when the heart rate exceeded 78.78 bpm, with the hazard ratio approaching 1 around this threshold (Figure 3).

### 3.6 Subgroup analysis of the interaction between heart rate and risk of death from cerebral infarction

**Figure 4:**
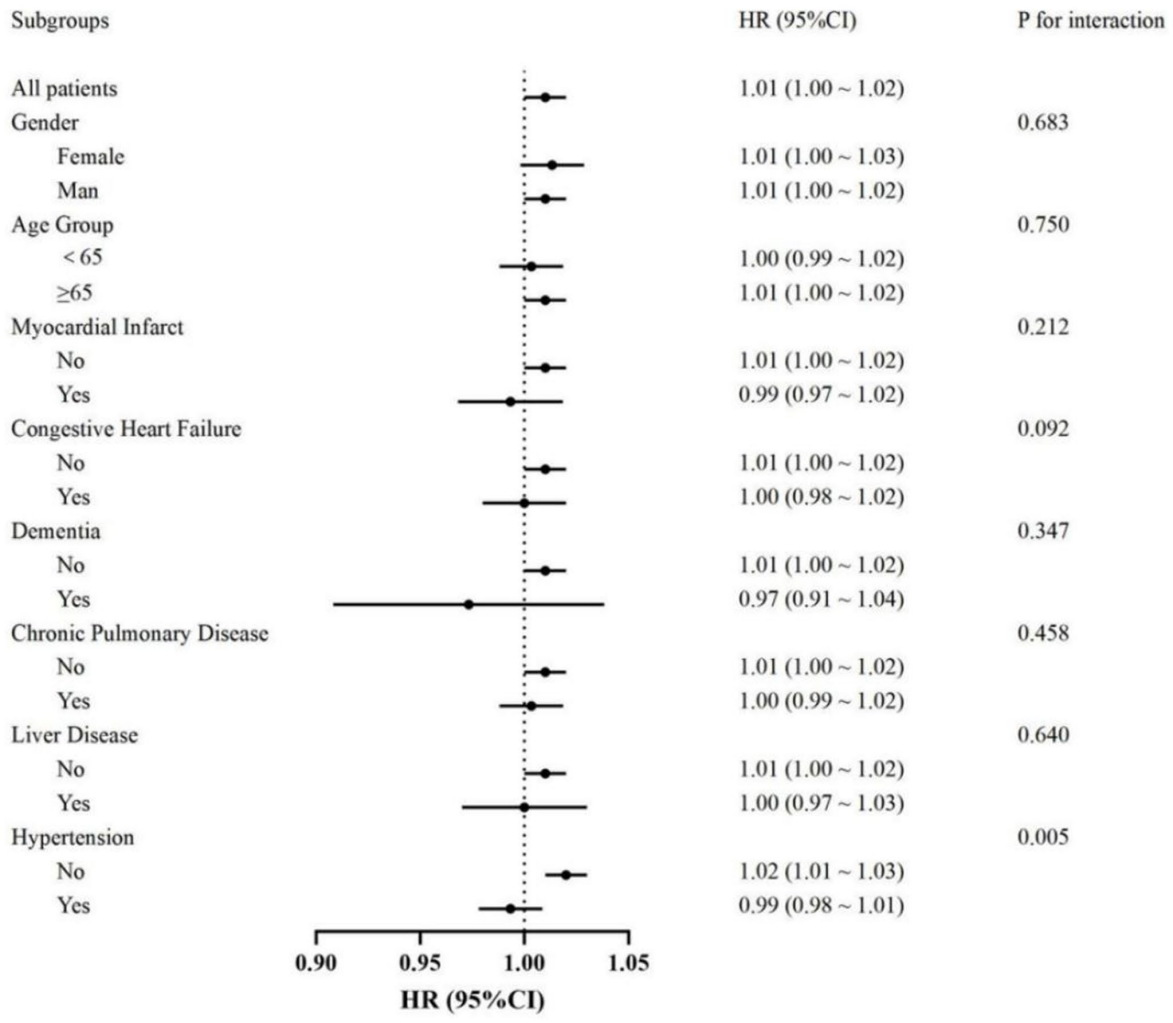
Forest plot showing hazard ratios (HR) and 95% confidence intervals (CI) for the association between heart rate on the probability of 28-day survival in patients with cerebral infarction, with the dotted line indicating the null (HR = 1).

The association between heart rate and in-hospital mortality was evaluated across various subgroups, including gender, age, myocardial infarction, congestive heart failure, dementia, chronic pulmonary disease, liver disease, and hypertension. Subgroup analyses demonstrated consistent associations between heart rate and mortality, with no significant interactions observed in most subgroups (all *p* for interaction >0.05). However, a significant interaction was identified in the hypertension subgroup (*p* for interaction = 0.005). Specifically, a significant association between heart rate and mortality was observed in patients without hypertension (HR: 1.02, 95% CI: 1.01–1.03), while no significant association was found in those with hypertension (HR: 0.99, 95% CI: 0.98–1.01) (Figure 4).

## 4 Discussion

Cerebral infarction is the most common type of stroke, characterized by a sudden interruption of blood flow to brain tissue, leading to neuronal damage. Clinical features include sudden weakness or numbness, typically on one side of the body, difficulty speaking or understanding speech, loss of vision in one or both eyes, dizziness, loss of balance, and unexplained severe headaches (16). Patients with large vessel occlusion tend to exhibit more pronounced infarct expansion during delayed treatment. Factors such as higher low perfusion intensity ratios and poorer collateral circulation are important predictors of this progression (17). Future efforts should focus on raising awareness, screening, and timely intervention to mitigate the impact of this debilitating disease.

When a cerebral infarction occurs, the blood supply to certain areas of the brain is interrupted, leading to hypoxia and neuronal death. The primary causes include embolic sources (such as atrial fibrillation), large artery atherosclerosis, and small vessel occlusion commonly associated with hypertension and diabetes (18, 19). From a pathophysiological perspective, cerebral infarction triggers a series of cascading events, including excitotoxicity, oxidative stress, inflammation, and blood–brain barrier disruption (20–22). Recent studies highlight the importance of biomarker identification to enhance early diagnosis and prognosis, with the aim of reducing the high mortality and disability rates associated with cerebral infarction (23).

This study found that higher heart rate was significantly associated with increased in-hospital mortality, with patients having heart rates >89.72 bpm showing significantly lower 28-day survival rates. Mortality risk increased when heart rate exceeded 78.78 bpm. Subgroup analysis indicated that heart rate was consistently associated with mortality in most subgroups, except for patients with hypertension, where no significant association was observed. Previous research has shown that patients with acute cerebral infarction who present with a higher initial heart rate at the time of hospitalization face a significantly greater risk of both all-cause mortality and cardiovascular death. This finding aligns with the results of our study, which highlights a strong association between heart rate indices and the prognosis of cerebral infarction patients. Therefore, monitoring and managing heart rate could play a crucial role in assessing prognosis and informing clinical decision-making (24, 25).

Heart rate plays a crucial role in predicting the prognosis of various diseases, particularly cardiovascular diseases and critical illnesses. An elevated resting heart rate is often associated with increased mortality and poor prognosis, especially in conditions such as heart failure and ischemic diseases (26). Advanced technologies, including interpretable machine learning models, have been developed to predict the prognosis of critically ill ICU patients by integrating heart rate with other physiological variables. For example, in ICU mortality prediction models, average heart rate, along with factors such as urine output and oxygen saturation, has been identified as a key predictive variable (27). In heart failure patients, improved heart rate control is associated with better recovery of ejection fraction and prognosis, highlighting its predictive significance in treatment outcomes (28) In addition, heart rate monitoring is being integrated into artificial intelligence and Internet of Things (IoT) platforms for continuous tracking, enabling real-time prognostic prediction and improving clinical decision-making in intensive care settings (26).

Heart rate significantly influences the prognosis of patients with cerebral infarction through various mechanisms. These abnormalities can exacerbate stroke-related complications. Autonomic dysregulation, characterized by excessive sympathetic nervous system activation and weakened parasympathetic modulation, is a key mediator in promoting pro-inflammatory states, endothelial dysfunction, and coagulopathy. For example, mechanisms that affect fibrosis and vascular remodeling, such as those involving SHARPIN protein, have been shown to impact stroke and cardiovascular outcomes. Reducing the expression of fibrosis-related proteins in cardiac tissue can alleviate stroke-related complications, highlighting the interplay between brain and heart health (29).

An elevated heart rate can impair the brain’s autoregulatory function by disrupting the balance between oxygen delivery and metabolic demand, thereby exacerbating ischemic damage in vulnerable brain areas. The mechanisms behind this phenomenon include increased oxidative stress, inflammation, and vascular dysfunction, which collectively lead to intensified neuronal injury. Research highlights that oxidative stress and mitochondrial damage are typically triggered by ischemia–reperfusion, playing a crucial role in disrupting autoregulatory mechanisms. For instance, inhibiting pathways such as JAK2/STAT3 and TLR9 can alleviate oxidative stress and improve prognosis by maintaining vascular stability (30). In addition, increased sympathetic nervous activity (often reflected in higher heart rates) may exacerbate ischemic damage by enhancing the release of pro-inflammatory cytokines and altering cerebral blood flow regulation. Therapies targeting inflammation and mitochondrial pathways, such as nicotinamide and ischemic preconditioning, have shown promise in restoring autonomic regulation and reducing ischemic injury (31). Monitoring and managing heart rate dynamics, including through pharmacological or non-invasive interventions such as heart rate variability biofeedback, can provide therapeutic benefits in stroke care, improving overall survival and functional recover. One study investigated the relationship between heart rate variability (HRV), neurological function, and clinical factors with mortality and behavioral functional outcomes in patients with ischemic stroke. The results suggest that HRV may be associated with 3-month behavioral functional outcomes (32).

Non-cardioembolic ischemic stroke is primarily caused by large artery atherosclerosis and small vessel occlusion, accounting for 70 to 85% of all ischemic strokes. We excluded patients with atrial fibrillation to specifically study the non-cardioembolic ischemic stroke population. Atrial fibrillation is a major cause of cardioembolic stroke and follows a distinct pathophysiological mechanism. The treatment strategies for cardioembolic and non-cardioembolic strokes differ significantly. Including atrial fibrillation patients in a study on ischemic stroke prognosis could introduce confounding bias due to differences in treatment approaches, thereby compromising the reliability of the study’s conclusions. Moreover, the relationship between heart rate and stroke prognosis differs in patients with atrial fibrillation. Atrial fibrillation patients exhibit greater heart rate variability, and the condition itself can lead to hemodynamic instability (33). Excluding patients with atrial fibrillation ensures greater homogeneity in the study population, making the research conclusions more specific and clinically relevant.

Studies have found that hypertension is one of the most significant risk factors for ischemic stroke (34). Hypertension significantly affects cerebral blood flow autoregulation and can lead to post-stroke hypoperfusion, inflammation, and cognitive impairment through mechanisms such as vascular smooth muscle dysfunction, blood–brain barrier disruption, and capillary rarefaction (35). Our study demonstrates that blood pressure significantly influences the relationship between heart rate and prognosis. In patients without hypertension, an elevated heart rate is significantly associated with worse outcomes in non-cardioembolic ischemic stroke. Based on clinical observations and previous research, we hypothesize that *β*-blockers or calcium channel blockers, which are frequently used by hypertensive patients, may reduce heart rate, thereby mitigating the adverse effects of heart rate elevation. Additionally, hypertension may lead to arterial stiffness and decreased vascular compliance, reducing the impact of heart rate on cerebral blood flow regulation. In contrast, non-hypertensive individuals may rely more on heart rate to maintain hemodynamic stability, making them more vulnerable to the detrimental effects of an elevated heart rate. Therefore, in ischemic stroke patients without hypertension, closer heart rate monitoring and potential heart rate control interventions should be considered to improve stroke prognosis.

This study used the MIMIC-IV database, which provides a large and diverse dataset that enables reliable analysis of clinical factors and prognosis in patients with cerebral infarction. However, it is important to recognize that the database has some inherent limitations. First, as a retrospective cohort study, the results are subject to inherent biases such as selection bias and unmeasured confounders. Although statistical adjustments have been made to minimize these biases, residual confounders cannot be completely excluded. Second, the MIMIC-IV database represents data from a single healthcare system, which may limit the generalizability of study results to other populations or healthcare settings. Differences in clinical practice, resource availability, and patient demographics may affect the external validity of study results. Third, the database lacks detailed neuroimaging data, such as CT or MRI findings, which are critical to accurately assess stroke severity, infarct size, and lesion location. The lack of these imaging parameters limits the ability to comprehensively assess the relationship between clinical variables and stroke prognosis. Finally, despite our efforts to address the problem of missing data through multiple interpolation methods, the presence of incomplete records and the potential bias introduced by estimation techniques remains a concern.

## 5 Conclusion

In conclusion, this study reveals a positive association between elevated heart rate and in-hospital mortality in patients with cerebral infarction without atrial fibrillation. Patients with heart rates greater than 90 bpm had significantly reduced 28-day survival, and this association remained consistent across most subgroups except for hypertensive patients. These findings suggest that heart rate may serve as an independent prognostic indicator of mortality in cerebral infarction patients. If further validated, this association could provide a rationale for developing therapeutic strategies targeting heart rate to improve clinical prognosis in this population.

## Data Availability

This study analyzed publicly available datasets, accessible at: https://physionet.org/content/mimiciv/2.0/.
